# The role of GTPase-activating protein ARHGAP26 in human cancers

**DOI:** 10.1007/s11010-021-04274-3

**Published:** 2021-10-30

**Authors:** Lingye Zhang, Anni Zhou, Shengtao Zhu, Li Min, Si Liu, Peng Li, Shutian Zhang

**Affiliations:** Department of Gastroenterology, Beijing Friendship Hospital, Capital Medical University, National Clinical Research Center for Digestive Diseases, Beijing Digestive Disease center, Beijing Key Laboratory for Precancerous Lesion of Digestive Diseases, Beijing, China

**Keywords:** ARHGAP26, GTPase-activating protein, Cancer, Rho GTPase

## Abstract

Rho GTPases are molecular switches that play an important role in regulating the behavior of a variety of tumor cells. RhoA GTPase-activating protein 26 (ARHGAP26) is a GTPase-activating protein and inhibits the activity of Rho GTPases by promoting the hydrolytic ability of Rho GTPases. It also affects tumorigenesis and progression of various tumors through several methods, including formation of abnormal fusion genes and circular RNA. This review summarizes the biological functions and molecular mechanisms of ARHGAP26 in different tumors, proposes the potential clinical value of ARHGAP26 in cancer treatment, and discusses current issues that need to be addressed.

## Introduction

RhoA GTPase-activating protein 26 (ARHGAP26) was first discovered in avian tissues by Hildebrand et al. in 1996 when they studied focal adhesion kinase (FAK) in integrin-mediated signaling. As such, this protein was initially named as GTPase regulator associated with FAK (GRAF) [1]. As a type of GTPase-activating protein (GAP), ARHGAP26 enhances the hydrolysis of GTPases and converts GTPases from an active form to an inactive form, thereby inhibiting signaling transduction [2]. Numerous studies have demonstrated that ARHGAP26 expression and involvement in tumorigenesis and tumor progression are not the same in different tumors. For example, ARHGAP26 expression is significantly reduced in acute myeloid leukemia (AML) [3], chronic myeloid leukemia (CML) [3, 4], and ovarian cancer [5], while the transcription factor activity of ARHGAP26 is significantly increased and expression of ARHGAP26 is upregulated in prostate cancer [6]. In gastric cancer, *ARHGAP26* is fused with claudin-18 gene (*CLDN18*), and the translated abnormal fusion protein regulates the development of gastric cancer [7–9], while circular RNA ARHGAP26 (circ-ARHGAP26) modulates microRNA through a “sponge” mechanism to affect the progression of gastric cancer [10–12]. In glioblastoma, ARHGAP26 acts as an important executive molecule downstream of the integrin complex to promote tumor invasion [13]. This article reviews the biological functions, molecular mechanisms, and clinical characteristics of ARHGAP26 in different tumors (Table 1), proposes its potential clinical applications, and explores the research directions and unsolved issues in related fields.

**Table 1:** The molecular mechanisms, functions, and clinical features of ARHGAP26 in human cancers

Cancer type	Molecular mechanism	Role	Biologic function	Clinical feature	Reference
Gastric cancer	CLDN18-ARHGAP26 fusion gene	Cancer promotor	Cell-ECM adhesion, proliferation, invasion, migration, stress fiber formation and clathrin-independent endocytosis	Pathological subtype, age, sex, tumor stage, OS, resistance to oxaliplatin and 5-fluorouracil	[7–9, 14–17]
	Circle ARHGAP26 RNA	Cancer promotor	Proliferation and cell apoptosis	Lymphatic metastasis	[11, 12, 27]
Myeloid malignancies	Low Expression of ARHGAP26	Unknown	Unknown	Complete remission rate, incidence of primary resistance disease, deaths in induction therapy, OS	[3, 4]
	Methylation of the ARHGAP26 promotor	Unknown	Unknown	Early event of AML development	[28, 29]
	MLL/ARHGAP26 fusion gene	Unknown	Unknown	Better response to treatment	[30–32]
Glioblastoma	Key downstream effector of CD151-α3β1 integrin complex signaling	Cancer promotor	Motility and invasion	Unknown	[13]
Prostate cancer	Transcription factor SP1 overactive	Unknown	Unknown	Unknown	[6]
Ovarian cancer	Low Expression of ARHGAP26	Cancer suppressor	Proliferation, migration, and invasion	OS	[5]

*CLDN18* claudin 18; *ARHGAP26* RhoA GTPase-activating protein 26, *ECM* extracellular matrix, *OS* overall survival, *AML* acute myeloid leukemia, *MLL* mixed lineage leukemia, *SP1* specificity protein 1

## Role of ARHGAP26 in tumors and its potential clinical value

### ARHGAP26 in gastric cancer

**Fig. 1:**
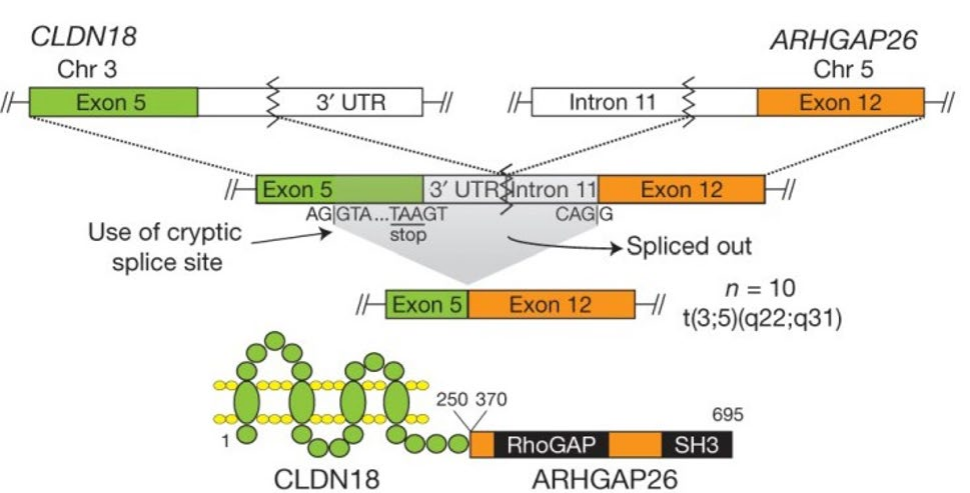
Schematic diagram of CLDN18-ARHGAP26 fusion gene and fusion protein. In the upper panel, the *CLDN18* gene is fused with the *ARHGAP26* gene, which initiates the translation of the fusion protein CLDN18-ARHGAP26 at a cryptic splice site. In the lower panel, the CLDN18-ARHGAP26 fusion protein contains the transmembrane domain of CLDN18 and the GAP and SH3 domains of ARHGAP26. CLDN18, claudin 18; ARHGAP26, RhoA GTPase-activating protein 26; *GAP* GTPase-activating protein, *SH3* src homology 3, *UTR* untranslated region

A study of 295 cases of gastric cancer in The Cancer Genome Atlas (TCGA) sample database showed that gastric cancer can be divided into four subtypes based on the molecular characterization. Expression of the *CLDN18-ARHGAP26* fusion gene was present in patients with a genomically stable subtype (Fig. 1) [7], which is the first report of the *ARHGAP26* fusion gene. Since then, multiple studies have shown the existence of this abnormal fusion gene [9, 14–16]. Based on research data, 151 out of 1908 (7.9%) patients with gastric cancer had this gene fusion [17]. Analysis of clinical characteristics showed that the *CLDN18-ARHGAP26* fusion gene is more common in female patients [9], young patients, and patients with diffuse-type gastric cancer (Lauren’s classification) [15] and is associated with poor prognosis [9, 14]. In vitro experiments have shown that the presence of the *CLDN18-ARHGAP26* fusion gene increases the migration and invasion ability of gastric cancer cells [8, 9, 15], as well as the resistance of tumor cells to chemotherapy drugs [9].

It is noteworthy that the high affinity of the CLDN18-ARHGAP26 fusion protein for the cell membrane, as well as its exclusive expression in gastric cancer cells provide new opportunities for targeted therapies [16]. CLDN18 protein is involved in the formation of tight junctions in epithelial cells [18]. A CLDN18 protein subtype, CLDN18.2, is the specific CLDN18 expressed in the stomach and is an important component of the gastric acid barrier [19–22]. Zolbetuximab, formerly ideal monoclonal antibody 362 (IMAB362), is a new type of immunoglobulin G1 antibody that is highly specific for the CLDN18.2 protein [22–24]. Thus, it has the potential to be used in targeted therapy for gastric cancer patients with the *CLDN18-ARHGAP26* fusion gene. This drug is currently undergoing clinical trials to evaluate its efficacy and safety [25, 26].

Through circular RNA microarrays, Shao et al. showed that the top ten upregulated circular RNAs in gastric cancer tissues included hsa_cirs_0074362, i.e., circ-ARHGAP26 (7.58-fold change, *p* = 0.01156) [27]. Normally, the *ARHGAP26* mRNA is transcribed from 46 kb of the genomic DNA at chromosome region 5q31.3, containing 23 exons. However, the circ-ARHGAP26 only contains the transcript from exon 5 to exon 11 in this chromosome region. Due to its structure, Xie et al. used divergent primers for circ-ARHGAP26 amplification and showed by RT-PCR that the expression of circ-ARHGAP26 was significantly lower in gastric cancer tissues than in paired normal adjacent tissues [12]. The expression of circ-ARHGAP26 was also lower in five gastric cancer cell lines (AGS, BGC-823, HGC-27, MGC-803, and SGC-7901) than that in the normal gastric epithelial GES-1 cell line. In addition, the expression of circ-ARHGAP26 in gastric tissues of patients with mild and moderate gastritis was also significantly lower than that of the normal gastric tissues, but higher than that of gastric cancer tissues [12]. In view of the contradictory results of these studies [12, 27], Lv et al. assessed the levels of circ-ARHGAP26 in gastric cancer cells and normal gastric epithelial cells and showed that circ-ARHGAP26 level was significantly lower in gastric cancer cell lines than in normal gastric epithelial cells [11]. In functional experiments, downregulation of circ-ARHGAP26 resulted in a decline in the proliferation of gastric cancer epithelial cells and an increase in apoptosis. Based on the above results, the expression of circ-ARHGAP26 in gastric cancer should be verified further. Meanwhile, more investigations are warranted to explore the underlying mechanism through which circ-ARHGAP26 affects tumorigenesis and progression of gastric cancer.

### ARHGAP26 in myeloid malignancies

Previous studies have shown that the expression of ARHGAP26 in AML was significantly lower than that in the control group [3, 4]. Further studies showed that AML patients with relatively high expression of ARHGAP26 have a longer overall survival. The expression of ARHGAP26 has no significant correlation with clinical characteristics, the French-American-British (FAB) classification, or cytogenetic risk subgroups of AML patients [3]. Similarly, the expression of ARHGAP26 in CML was also significantly lower than that in the controls. In addition, the expression of ARHGAP26 significantly decreased with the progression of CML. No significant difference in the expression of ARHGAP26 was found between CML patients in the remission phase and chronic phase [4]. In terms of the mechanism, a significant increase in the methylation ratio of the *ARHGAP26* promoter in patients is an important reason for the decrease in ARHGAP26 expression [28]. Another study showed that the transcription level of *ARHGAP26* was significantly lower in AML patients compared with the normal population, regardless of whether the *ARHGAP26* promoter was methylated. In addition, patients with a methylated *ARHGAP26* promoter had a lower *ARHGAP26* transcription level, indicating that hypermethylation of the *ARHGAP26* promoter was an early event in AML progression [29].

Gene fusion is a common chromosomal structural abnormality in patients with acute leukemias. The majority of translocations that occur at 11q23 disrupt mixed lineage leukemia gene (*MLL*) and fuse it to many different partner genes [30]. Studies have shown that the *MLL/ARHGAP26* fusion is a chromosomal abnormality in infants and young children with AML. Through case summary analysis, it was found that infants with the *MLL/ARHGAP26* fusion responded well to treatment [30–32].

### ARHGAP26 in glioblastoma

Studies have shown that the integrin complex CD151-*α*3*β*1 significantly promotes the invasion and migration of glioblastoma, and ARHGAP26 is a key molecule downstream of the complex [13]. Moreover, antibodies against ARHGAP26 were first discovered in patients with subacute cerebellar ataxia [33, 34]. Subsequent studies have shown that these antibodies are related to cognitive impairment and dyslexia [35–38]. Interestingly, researchers also detected other systemic tumors in patients with such neurological diseases who were positive for ARHGAP26 antibodies, suggesting that anti-ARHGAP26 antibodies may be a potential tumor predictor in such patients [38, 39]. Nevertheless, the role that these antibodies play in the tumorigenesis of other cancers remains unclear. Large-scale clinical trials should be carried out to investigate the value of using anti-ARHGAP26 antibodies to identify patients with the aforementioned characteristics.

### ARHGAP26 in prostate cancer

An analysis of different genes in a cohort from the Gene Expression Omnibus (GEO) database, which contained 18 prostate cancer tissues and 21 normal tissues, showed that the activity of transcription factor specificity protein 1 (SP1), which significantly upregulates the expression of ARHGAP26, was significantly upregulated in prostate cancer [6]. However, no further in vivo or in vitro studies were conducted to clarify the impact of ARHGAP26 on the phenotype of prostate cancer. In addition, the correlation between the clinicopathological characteristics of prostate cancer patients and upregulation of ARHGAP26 expression is also worth exploring.

### ARHGAP26 in ovarian cancer

A previous study showed that expression of ARHGAP26 in ovarian cancer tissues was significantly reduced and related to poor prognosis of the patients [5]. Cytological experiments have shown that upregulation of ARHGAP26 leads to a decrease in cell proliferation, migration, and invasion and downregulation of the downstream molecules, including GTP-RhoA, *β*-catenin, vascular endothelial growth factor (VEGF), matrix metallopeptidase (MMP)2, and MMP7, whereas downregulation of ARHGAP26 leads to the opposite cellular phenotypes—all of which can be inhibited by the *β*-catenin inhibitor, DKK1. Animal experiments have shown that upregulation of ARHGAP26 reduces the lung metastasis of ovarian cancer cells. Interestingly, upregulation of ARHGAP26 in SKOV3 ovarian carcinoma cells was shown to effectively inhibit the migration and invasion of tumor cells due to the upregulation of smad ubiquitination regulatory factor 1 (SMURF1). As an E3 ubiquitination ligase, SMURF1 degrades ARHGAP26 through the ubiquitination pathway. Thus, researchers believe that SMURF1-dependent regulation of ARHGAP26 ubiquitination promotes the invasion and migration of ovarian cancer cells through the *β*-catenin pathway [5].

## Mechanism of ARHGAP26 in cancers

**Fig. 2:**
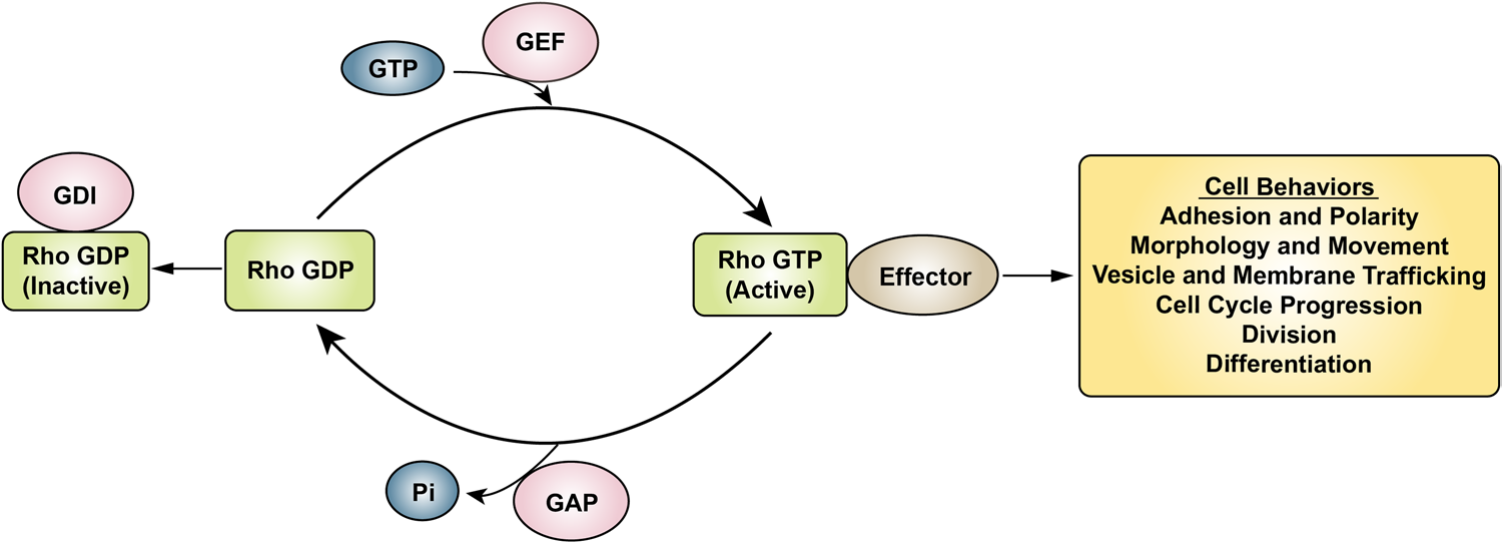
The cycle of the GTP-binding and GTP-hydrolysis and signaling functions of Rho GTPases involved in cells. The schematic shows how GEFs, GAPs, and GDIs regulate the cycle of the Rho GTPase signaling pathway and the associated cell behaviors. *GEF* guanine nucleotide exchange factor, *GAP* GTPase-activating protein; *GDI* Rho GDP dissociation inhibitor

Rho GTPases are a group of signaling proteins belonging to the Ras GTPases superfamily [40, 41]. There are 22 types of mammalian Rho GTPases [42]. Among them, RhoA, rac family small GTPase 1 (Rac1), and cell division cycle 42 (Cdc42) are the most commonly studied. Rho GTPases are molecular switches that regulate signaling pathways through GTP-loading/GTP-hydrolysis (Fig. 2), namely GAPs, guanine nucleotide exchange factors (GEFs), and Rho GDP dissociation inhibitors (GDIs) [43]. GAPs promote GTPase hydrolysis to inhibit signaling transduction. GEFs catalyze the GTPase-loading reaction to stimulate signaling transduction, and GDIs inhibit the dissociation of GDP by binding to GDP-bound Rho GTPase, thereby inhibiting the activation of signaling pathways [43, 44]. Rho GTPases play an important role in cellular processes, including cell adhesion and polarity, cell morphology and movement, exchanges between vesicles and cell membranes, cell cycle, cell division, and cell differentiation (Fig. 2) [44–46]. In addition, Rho GTPases are closely related to tumorigenesis and tumor progression.

ARHGAP26 is a GAP, and it is specific for only Cdc42 and Rho A [1, 2]. ARHGAP26 converts both proteins from the GTP to GDP forms, which inactivates them, thereby regulating downstream molecules [44]. The ARHGAP26 molecule includes the GAP domain in the central region, the src homology 3 (SH3) domain in the C segment, and the Bin/amphiphysin/Rvs (BAR) and pleckstrin homology (PH) domains at the N-terminal. These domains are highly conserved among GAPs and GEFs [47, 48]. The GAP domain mainly inactivates Rho GTPases by enhancing the hydrolysis of GTPases [1, 2]. The SH3 domain is combined by a variety of regulatory molecules to regulate the activity of the GAP domain of ARHGAP26 [1, 2, 49]. The BAR and PH domains of ARHGAP26 regulate endocytosis by binding and inducing membrane curvature [50–53]. Numerous studies have shown that Rho GTPases play important, but different roles in a variety of tumors [54–56]. Thus, this partly explains the difference in the role of their upstream regulatory molecule, ARHGAP26, in different tumors.

Abnormal fusion of *ARHGAP26* with other genes was first reported in 2000 [30]. *ARHGAP26* was fused to *MLL* in a unique t (5; 11) (q31; q23) fusion in infants with myelodysplastic syndrome (MDS) and AML. Since then, several cases with similar fusion genes have been reported; however, *MLL/ARHGAP26* fusion is relatively rare in patients with hematological malignancies [31, 32]. In 2014, the fusion of *ARHGAP26* and *CLDN18* (*CLDN18-ARHGAP26* fusion) was first reported in gastric cancer patients [7]. There are many types of *CLDN18-ARHGAP26* fusion. Among them, *CLDN18*/exon5-*ARHGAP26*/exon12 fusion is the most common [17]. The *CLDN18-ARHGAP26* fusion translates into the CLDN18-ARHGAP26 fusion protein by activating a cryptic splice site within exon 5 of *CLDN18* (Fig. 1) [14, 15, 18]. The fusion protein retains the GAP domain located in the central region of ARHGAP26 and the SH3 domain located at the C-terminal [8, 17], due to which the fusion protein retains some functions, including regulating its downstream RhoA pathway and mediating the integrin signaling pathway [1, 2, 49]. The N-terminal BAR and PH domains of wild-type ARHGAP26 are missing from the fusion protein, so the GAP domain activity of the fusion protein cannot be regulated by its upstream molecules, and the ability of wild-type ARHGAP26 to regulate the endocytosis pathway is also affected [51, 57–59]. CLND18 protein is involved in the formation of tight junctions in epithelial cells and plays a crucial role in the gastric acid barrier [19–22]. The CLDN18-ARHGAP26 fusion protein only has the transmembrane domain of CLDN18 and lacks the cytoplasmic part at the C-terminus. Therefore, the adhesion function of epithelial cells expressing the fusion protein is impaired, which, in turn, leads to dysfunction of the gastric mucosal barrier [7, 8, 16, 17, 21]. The two abnormal functions of the fusion protein that promote the invasion and migration of gastric cancer cells also partly explain the decreased survival rates and worse prognosis of patients with diffuse gastric cancer with the *CLDN18-ARHGAP26* fusion compared with those without the *CLDN18-ARHGAP26* fusion.

Circ-ARHGAP26 was first discovered in human gastric cancer tissues by Shao et al. in 2017 [27]. Circular RNA, also known as circRNA, is a long-chain, non-coding precursor RNA (pre-RNA) formed by non-canonical splicing. Its most important biological function is to regulate gene expression [60]. Post-transcriptional regulation has been an extensively recognized method for circRNA to regulate gene expression, (i.e., using circRNA as a “microRNA sponge” or “microRNA reservoir” for sequestering and regulating microRNA) [61, 62]. Studies have shown that regulation of circRNA plays an important role in tumorigenesis and tumor progression [60, 63]. Moreover, it has been reported that circ-ARHGAP26 is closely related to the expression of the tumor marker carbohydrate antigen 19–9 (CA19-9) [12], which may provide evidence for the involvement of circ-ARHGAP26 in the expression and regulation of tumor-related proteins.

## Issues to be solved

First, the upstream regulators responsible for the abnormal expression of ARHGAP26 in tumors are the most important issues that need to be addressed. The regulatory mechanisms of ARHGAP26 in different tumors vary, including changes in the expression level of ARHGAP26, abnormal fusion of *ARHGAP26* with other genes, and microRNA sponge mechanism of circ-ARHGAP26. Understanding the cause of the abnormal expression of ARHGAP26 aids in evaluation of the tumor risk and implementation of pre-intervention.

Second, downstream mechanisms responsible for the abnormal expression of ARHGAP26 in tumors still need to be further investigated, including the expression of circ-ARHGAP26 in gastric cancer patients. The mechanism of regulation of microRNAs by circ-ARHGAP26 to alter the expression of downstream genes still needs to be explored. The underlying mechanism of the effects of *CLDN18-ARHGAP26* fusion protein on the downstream molecules to cause tumorigenesis and progression of gastric cancer also needs to be further studied.

Lastly, although the clinical trials using the Zolbetuximab monoclonal antibodies against CLDN18.2 have reported encouraging results [25, 26], there are still problems that need to be resolved. A question worth exploring is whether *CLDN18-ARHGAP26* fusion has a positive correlation with the CLDN18.2 protein expression. The answer to this question will determine whether this fusion gene can be used as a specific indicator of tumors that would be sensitive to Zolbetuximab-targeted therapy [17]. In addition, given the differences between the Eastern and Western populations, Zolbetuximab in combination with other chemotherapies exerts a greater toxicity in Asian people than in other populations [22]. Therefore, the tolerability and therapeutic effect of the combination of Zolbetuximab and other chemotherapeutics in the Asian population still needs to be evaluated.

## Conclusion

**Fig. 3:**
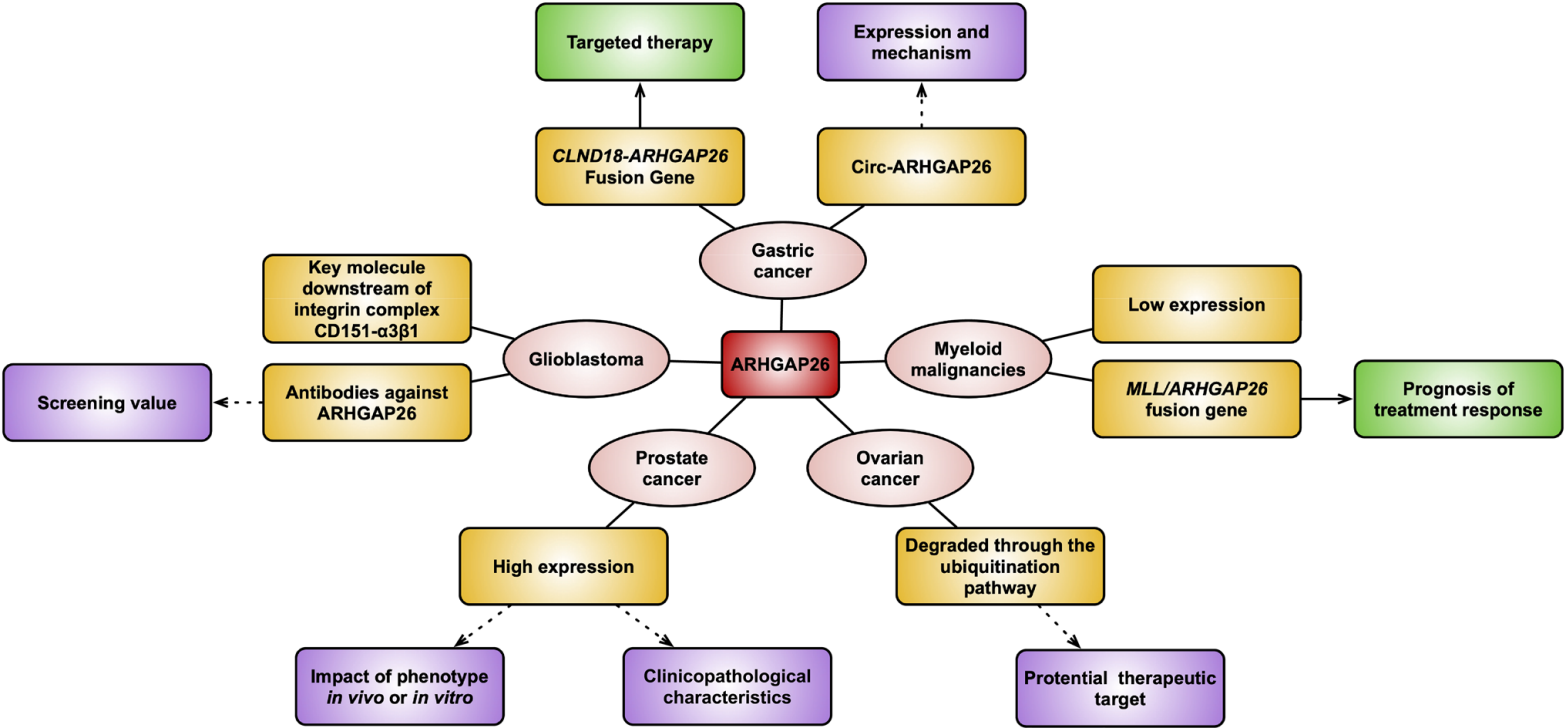
Summary diagram of the role of ARHGAP26 in human cancers. The diagram summarizes the expression level and patten of ARHGAP26 in different human cancers (yellow), potential clinical issues (green), and questions needed to be solved (purple)

ARHGAP26, a GAP regulating the Rho GTPases, plays a crucial role in tumorigenesis and progression of human cancers. The expression level and pattern of ARHGAP26 in various tumors are quite different. However, for a given type of cancer, it can be used as a biomarker because of its altered expression compared with normal tissues or cells. Moreover, the CLDN18-ARHGAP26 fusion protein may became a novel target for the treatment of gastric cancer patients in the future. Notably, although many studies have explored the biological functions and molecular mechanisms of ARHGAP26, there are still many contradictions and problems worth exploring (Fig. 3). Therefore, more basic experiments and large-scale clinical trials are needed in this field.

## Data Availability

All data were included in the manuscript.
